# Sample Preparation Methods for Lipidomics Approaches Used in Studies of Obesity

**DOI:** 10.3390/molecules25225307

**Published:** 2020-11-13

**Authors:** Ivan Liakh, Tomasz Sledzinski, Lukasz Kaska, Paulina Mozolewska, Adriana Mika

**Affiliations:** 1 Department of Pharmaceutical Biochemistry, Medical University of Gdansk, Debinki 1, 80-211 Gdansk, Poland; liakh_ivan@mail.ru (I.L.); tomasz.sledzinski@gumed.edu.pl (T.S.); paulina.mozolewska@gumed.edu.pl (P.M.); 2 Department of Toxicology, Medical University of Gdańsk, Al. Gen. Hallera 107, 80-416 Gdańsk, Poland; 3 Department of General, Endocrine and Transplant Surgery, Faculty of Medicine, Medical University of Gdansk, Smoluchowskiego 17, 80-214 Gdansk, Poland; lukasz.kaska@wp.pl; 4 Department of Environmental Analysis, Faculty of Chemistry, University of Gdansk, Wita Stwosza 63, 80-308 Gdansk, Poland

**Keywords:** sample preparation, obesity, lipids, protein precipitation, liquid–liquid extraction, solid-phase extraction, biological samples

## Abstract

Obesity is associated with alterations in the composition and amounts of lipids. Lipids have over 1.7 million representatives. Most lipid groups differ in composition, properties and chemical structure. These small molecules control various metabolic pathways, determine the metabolism of other compounds and are substrates for the syntheses of different derivatives. Recently, lipidomics has become an important branch of medical/clinical sciences similar to proteomics and genomics. Due to the much higher lipid accumulation in obese patients and many alterations in the compositions of various groups of lipids, the methods used for sample preparations for lipidomic studies of samples from obese subjects sometimes have to be modified. Appropriate sample preparation methods allow for the identification of a wide range of analytes by advanced analytical methods, including mass spectrometry. This is especially the case in studies with obese subjects, as the amounts of some lipids are much higher, others are present in trace amounts, and obese subjects have some specific alterations of the lipid profile. As a result, it is best to use a method previously tested on samples from obese subjects. However, most of these methods can be also used in healthy, nonobese subjects or patients with other dyslipidemias. This review is an overview of sample preparation methods for analysis as one of the major critical steps in the overall analytical procedure.

## 1. Introduction

Obesity remains one of the pressing problems of modern society, therefore, studies of the mechanisms underlying its occurrence and the therapies used to treat it continue to be relevant. Depending on the hypothesis, a wide range of research methods can be used, ranging from purely assessing biochemical parameters to deep psychological research. However, research usually involves standard procedures such as measuring body mass index (BMI) or fat content in human subjects. Among the biochemical parameters, those that undergo the greatest changes with obesity (lipid profile, fasting glucose, insulin, etc.) are first examined.

A large amount of accumulated data on obesity allows for their meta-analysis and underlies a large number of systematic and retrospective reviews [1]. In particular, studies related to bariatric surgery are a powerful source of data because these types of surgery involve altering the stomach, intestines, or both to induce weight loss [2,3,4,5,6,7]. In addition, a large number of studies of obesity are associated with cardiovascular diseases [8,9,10,11], diabetes and metabolic syndrome [12,13,14]; many studies are in the field of diets, psychology and neurology [15,16,17,18,19,20,21,22,23].

**Figure 1:**
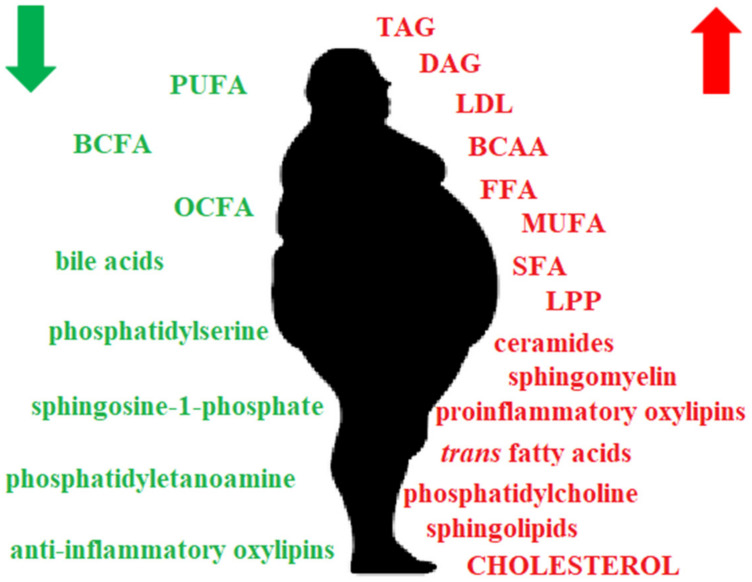
Lipid alterations in obesity. Lipids in red are elevated in obesity, and lipids in green are reduced. BCAA—branched chain amino acids; BCFA—branched chain fatty acids; DAG—diacylglycerols; FFA—free fatty acids; HDL—high density lipoproteins; LDL—low density lipoproteins; LPP—lipid peroxidation products; MUFA—monounsaturated fatty acids; OCFA—odd chain fatty acids; SFA—saturated fatty acids; PUFA—polyunsaturated fatty acids; TAG—triacyclglycerols.

While the number of parameters in the study of obesity itself is limited only by the imagination of scientists and the equipment available in the laboratory [24,25,26,27,28,29], the study of obesity in connection with other diseases is strictly subordinate to the study area and is often limited to several parameters, such as BMI and total fat content. [30,31,32,33]. In addition, determining the diagnosis of obesity is always primary in that work; for this purpose, the most commonly used method is the calculation of BMI. The World Health Organization used BMI to categorize humans into underweight (< 18.5), normal weight (18.5–24.9), overweight (25–29.9) and obese (BMI ≥ 30) categories [34]. Since BMI may not be a good indicator of obesity for bodybuilders and other groups of athletes [35,36], body fat [37,38,39] and total body water [40,41] can be determined in these groups, as can concomitant states of lipid alterations in blood (dyslipidaemia) [42], hyperinsulinemia [43], etc. Since obesity is directly related to lipid metabolism, it is interesting to study not only standard plasma parameters but also alterations in specific lipid groups in serum [44]. However, due to the much higher lipid accumulation in obesity (Figure 1) and many alterations in the lipid composition, the methods used for sample preparations for lipidomic studies in samples from obese subjects sometimes have to be modified. Thus, the purpose of this review was to collect and systematize information on sample preparation for lipid analysis in the study of obesity. We focused on both standard procedures and new promising methods. However, most of these methods can be also used in healthy, nonobese subjects or patients with other dyslipidemias.

In the study of obesity, determining triglyceride (TG) and cholesterol levels is of great clinical importance. Basic blood test results for total cholesterol (TC), TG and cholesterol in lipoprotein fractions (low density lipoproteins (LDL) and high density lipoproteins (HDL)) should be considered together. To study these indicators and related and often required for standard clinical practice indicators (C-reactive protein, glucose, insulin levels, etc.), there are many standard methods and their modifications that make these analyses routine in clinical practice, and they will not be presented in this review because of their routine nature. This work is focused more specifically on lipid groups, the study of which is carried out much less frequently because it requires the use of complex extraction methods and/or high-performance equipment. However, the alterations found in the extended lipidome may provide much more additional information about metabolic disorders in obese subjects than standard lipidograms.

## 2. Methods of Sample Preparation for Lipidomic Studies

### 2.1. Sample Collection and Storage

Fat metabolism disorders are detected by determining the lipid spectrum of the blood. Blood for a study is taken from a vein, always on an empty stomach (12–14 h after eating); otherwise, the results of the study are distorted, since 1-4 h after eating, alimentary hyperlipaemia occurs [45]. During blood sampling, adverse events such as haemolysis, coagulation, and platelet activation should be avoided, but the class of anticoagulants used should also be taken into account since calcium-chelating coagulants (ethylenediaminetetraacetic acid (EDTA) and citrate) can cause the calcium-dependent formation or degradation of certain classes of lipids ex vivo [46].

Different classes of lipids are subject to different changes during storage. Long-term storage of plasma at room temperature (RT) leads to an increase in lysophosphatidylethanolamines (LPE), lysophosphatidylcholines (LPC) and fatty acids (FAs), while phosphatidylethanolamines (PE) and phosphatidylcholines (PC) decrease, which suggests the breakdown of ester bonds in these phospholipids [47]. Avoiding freeze-thaw cycles is no less important because with their increase, the number of lipid metabolites decreases significantly [48].

### 2.2. Pre-Extraction Additives

Additives used during or before extraction serve a variety of purposes. Internal standards are a measure of extraction efficiency. In many cases, lipidomic studies of obesity are accompanied by the determination of obesity-associated hormone levels (such as ghrelin, obestatin, glucagon, leptin, and adiponectin); therefore, protease inhibitor cocktails are added to serum/plasma samples to increase hormone stability [49]. In addition, various detergents serve to facilitate cell destruction during homogenization, and buffers are used to maintain a stable pH. The most commonly added substances to prevent oxidative processes during extraction are antioxidants and radical scavengers such as butylated hydroxytoluene (BHT) [50,51]. This is especially important when studying unstable compounds such as oxylipins [52,53,54], which are the metabolites of polyunsaturated fatty acids.

### 2.3. Sample Stability

It is generally assumed that lipids are highly stable at RT, while it is advisable to not allow them to overheat during homogenization and to prevent oxidation by the addition of antioxidants [55]. Despite this, many studies consider the effects of storage conditions, the number of freeze/thaw cycles and the behaviour of organic compounds in experimental conditions. Jiang et al. validated a liquid chromatography-tandem mass spectrometry (LC-MS/MS) method for the determination of ceramides (Cer) in human plasma and determined the stability of each analyte at low- and high-quality control concentrations under long-term storage (39 days at -80 °C), freeze/thawing (five times), tabletop mode (14 h at RT before sample extraction) and autosampler conditions (3 days). The results showed that Cer (22:0) and Cer (24:0) were stable in human plasma under all conditions [56]. Ferreiro-Vera et al. assessed the stability of eicosanoids in serum under experimental conditions; every hour for 8 h, they analysed samples spiked with eicosanoids, and no significant differences in analyte concentrations were found [57]. Zeng and Cao also showed sufficient stability of short-chain FAs (SCFAs) and ketone body derivatives during autosampler storage (5 °C; 48 h), after 2 h at RT and after three freeze/thaw cycles [58]. Klawitter et al. showed that freeze/thaw cycles and long-term storage of plasma (6 h; RT) should be avoided to prevent changes in the composition of lipid classes of very low-density lipoprotein (VLDL) (loss of cholesterol esters and phospholipids), while free fatty acid (FFA) concentrations did not change under the same conditions [59]. Oxylipins are especially unstable in this regard, and improper collection and storage of samples can lead both to a significant decrease in their level and to an increase in their content due to enzymatic and non-enzymatic oxidation [60]. Some oxylipins (resolvins and prostanoids) are unstable even at −20 °C [61], so the manufacturers of their standards recommend storing them at −80 °C, while the concentration of prostaglandins can significantly decrease with prolonged storage at even −80 °C [62].

### 2.4. Extraction Methods

#### 2.4.1. Protein Precipitation

Protein precipitation (PPT) is used to remove protein from samples, therefore, when carrying out PPT during sample preparation, it is important that the chosen solvent causes protein denaturation and, at the same time, is a good solvent for lipids [55]. In addition, precipitation of proteins that make up a large volume of the analysed matrix is necessary since some groups of lipids are present in the matrix in trace amounts. This helps to minimize the risk of lack of detection or misidentification and to release protein-bound compounds prior to target lipid extraction [63]. Most often, PPT is preceded by subsequent solid-phase extraction (SPE) and liquid-liquid extraction (LLE). However, in the cases using high-performance equipment or shotgun lipidomics, PPT alone may be sufficient. Bellissimo et al. studied the metabolic profiles of obese individuals and used plasma acetonitrile (ACN) PPT before liquid chromatography-high-resolution mass spectrometry (LC-HRMS) analysis [64]. Jiang et al. when developing a method for validating Cer in human plasma, optimized the PPT method using 3 organic solvents (methanol (MeOH), ACN and isopropanol (IPA)) separately and in combination with chloroform. The mixture IPA:chloroform (9:1) was the most effective [56]. Drotleff et al. performed PPT during lipidomics of murine plasma by adding 55 µL of IPA and 20 µL of MeOH to 25 µL of plasma [65]. Söder et al. performed liquid chromatography-time-of-flight mass spectrometry (LC-TOF-MS)-based metabolomics analysis in overweight dogs using methanol extraction (5 μL of plasma:495 μL of MeOH), which allowed the determination of 317 phospholipids in the plasma samples [66].

#### 2.4.2. Liquid–Liquid Extraction

The high solubility of the hydrocarbon chains of lipids in organic solvents allows the use of LLE for the separation of lipids in various immiscible liquids. Widely used methods such as those of Folch [67] and Bligh and Dyer [68] have the drawback of using toxic solvents [69]; in addition, some classes of lipids (for example, lysophospholipids (LPL)) can remain in the aqueous phase [70]; however, many proposed modifications of these methods can overcome the above disadvantages, and these methods are still widely used in lipidomics of obesity samples [70,71,72,73,74,75,76]. Methyl tert-butyl ether (MTBE) extraction, which has been popular recently, is undergoing various modifications and shows very good efficiency over classical methods [69]. In the study of obesity, MTBE extraction is used to isolate lipids from liver tissue [77], skeletal muscle [78], adipose tissue [79] and plasma [50,72].

#### 2.4.3. Solid-Phase Extraction

SPE is more suitable than LLE for target lipidomics because it allows fractionation of specific lipid classes after LLE [80,81]. Therefore, in a lipidomics study, SPE is resorted to when it is necessary to isolate specific lipid groups or species that are present in the sample in a small amount, such as eicosanoids [57], LPL [70], oxidized phospholipids [75], serum sterols [82] oxysterols, endocannabinoids, and Cer [83], non-esterified FA and oxylipins [53,84,85,86]. Due to the wide variety of SPE protocols and commercially available SPE columns, there are studies in which these parameters are compared, for example, in studies of fatty acid esters of hydroxy fatty acids (FAHFAs) in serum [87] or oxylipins in human plasma [88,89]. Additionally, SPE helps to separate lipids in complex matrices with a large lipid abundance, such as adipose tissue [83,90] or brain tissue [80].

Since most of the extraction protocols described below are based on the SPE and LLE methods, the question arises—which of these protocols is more suitable for lipidomics studies. The advantages and disadvantages of PPT, LLE and SPE have been reviewed comprehensively elsewhere [91,92,93,94,95,96] and discussions on which of the methods is better are still ongoing. Among the main advantages of the LLE, one can note its low costs and the presence of a large number of well-established protocols. In turn, SPE is assumed to have a less pronounced matrix effect, there is less transfer of the aqueous phase, less toxic solvents are used, and it is less labour and faster. However, the last 2 statements are controversial [91]. On the other hand, the advantage of SPE, such as the high selectivity can be considered a disadvantage in the case when it is necessary to separate simultaneously several analytes with different physical and chemical properties which would require several different SPE columns. In addition, the complex structure of sorbents in SPE columns increases the risk of differences between individual bathes, in contrast to LLE, which uses highly purified simple organic solvents. It can be difficult to achieve high recovery efficiency with SPE since some analytes may partially elute in a different fraction during separation. However, despite the complication of the process and sample preparation time, the most comprehensive results can be obtained by combining LLE with SPE [70,79,80,83,87,97,98,99,100,101] or with TLC [74,86,102,103,104] approaches in one study.

#### 2.4.4. Other Extraction Methods

In addition to the well-established routine extraction methods described above, such as LLE, SPE, and PPT, also more modern but at the same time rarer extraction methods are used in the studies of obesity, such as solid-phase microextraction (SPME), stir bar sorptive extraction (SBSE), dispersive liquid–liquid microextraction (DLLME) and their variants. The main disadvantage of the above-listed solvent extraction is the use of organic solvents that have such disadvantages as toxicity and harmfulness to the environment. In addition, they must be of high purity, which increases the cost of analysis [105]. However, SPME, SBSE, DLLME are a solvent-free sample preparation method that is easy to use, does not require preliminary sample preparation, and is easily automated [94].

The most widely used technique of above mentioned is SPME. In combination with gas chromatography-mass spectrometry (GC-MS) it can be used not only for analysis of volatile organic compounds, but also for the extraction of fatty acids and fatty acid esters from solid tissues and biofluids, which requires a very small sample volume and reduces the matrix effect [106]. Although SPME can be used for lipidomics studies, in obesity studies these methods are also used to study non-lipid compounds. SPME followed by GC/MS was used to analyze aroma compound headspace release from extra virgin olive oil after the interaction of saliva in obese and overweight individuals [107], to evaluate volatile organic compounds of gut microbiota of obese patients [108,109], and for urinary volatile organic compounds profiling in overweight children [110].

The SBSE method, like SPME, is a method of sample preparation without the use of solvents and with the use of a solid sorbent for preliminary concentration of the analyte before analysis. The surface area of the sorbing polymer is greater in SBSE than in SPME [111]. Eslami et al. used SBSE followed by HPLC for quantification of ghrelin in human plasma [112].

The DLLME method is based on the rapid mixing of dispersing and extraction solvents with an aqueous sample, resulting in the formation of an emulsion consisting of fine particles of the extraction solvent dispersed in the aqueous phase, then the solvent is separated from the sample by centrifugation [113]. Amin et al. used DLLME following GC/MS method for the evaluation of urinary Bisphenol A in obese subjects [114]. Krawczyńska et al. applied DLLME technique for the determination of vitamin D in obese patients plasma [115].

Thus, the relatively small number of studies in lipidomics using above methods is explained by their recent appearance, while such advantages as relative easiness of implementation, accuracy, small sample volume and lack of organic solvents make these extraction methods promising.

## 3. Preparation of Different Sample Types

Most methods of lipid extraction from biological samples are based on the dissolution of hydrocarbon chains in organic solvents mixed in various combinations (Folch [67] and Bligh and Dyer [68] and modifications of their method). However, there is no unified methodology, and the specific protocol should be selected depending on the lipid class being studied. Especially in studies in obese subjects, when the amounts of some lipids are elevated, others are present in trace amounts and obese subjects have some specific alterations of the lipid profile, it is best to use a method previously tested on samples from obese subjects. Table 1 summarizing sample preparation methods for lipidomics approaches described below is located at the end of this section.

### 3.1. Serum/Plasma Lipids

**Figure 2:**
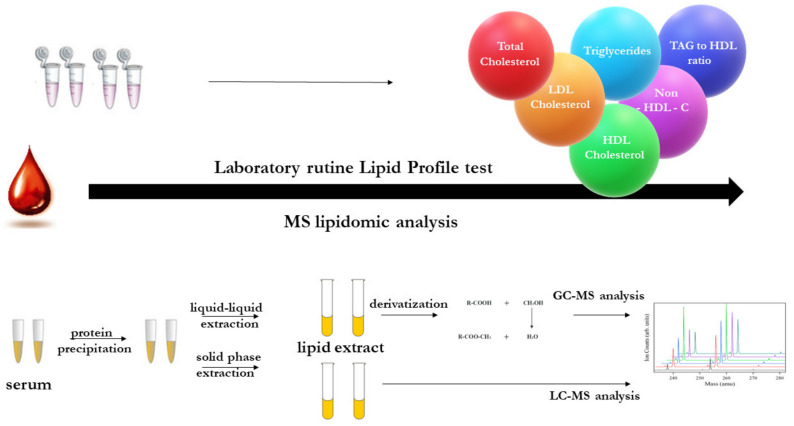
Lipid analysis in diagnostic laboratory vs. advanced lipidomic studies.

Plasma is the medium most responsive to changes in the body, which makes it one of the most important sources of information. However, at the same time, a large number of metabolites in the plasma and the fast rate of variation in their content can make it difficult to find specific markers of diseases. Most often, in the study of obesity, an important parameter such as the fasting lipid profile, which includes TC, HDL cholesterol, LDL cholesterol and TG, is determined for blood plasma [116]. A powerful indicator may be the content of FFAs in the blood, which reflects the influx of excess FAs from visceral fat into the liver, which is observed in obesity [117,118]. However, advanced approaches using lipidomics based on mass spectrometry can identify hundreds of lipid species belonging to dozens of lipid classes in plasma [119,120] (Figure 2).

Blewett et al. used the modified Folch method to extract lipids from the plasma of obese rats, after which phospholipids were separated on silica G plates; for this, they were visualized using 8-anilino-1-naphthalenesulfonic acid under ultraviolet (UV) light and compared with the corresponding standards. The resulting phospholipid silica band was removed, and FA methyl esters (FAMEs) were prepared for further analysis by gas chromatography (GC) [121]. Additionally, Choromańska et al. used a combination of LLE and thin-layer chromatography (TLC) to extract lipid fractions (FFA and triacylglycerols (TAG)) from plasma in women with morbid obesity. Lipid fractions were extracted from 200 μL of plasma samples according to the method of Bligh and Dyer. Then, the samples were separated by TLC on silica gel plates (Silica Plate 60, 0.25 mm; Merck, Darmstadt, Germany). The separation was carried out in a solvent containing heptane, isopropyl and acetic acid (60:40:3; *v*/*v*/*v*) after methylation with boron in trifluoride (BF3)-MeOH. Than samples were analysed by gas chromatography-mass spectrometry (GC-MS) [74].

Analysis of acyl-lysophosphatidic acids (LPAs) is of clinical importance in terms of prevention of obesity [122]. Yoon et al. established a method of LPA determination in human plasma. For this, extraction was performed with a MeOH/chloroform mixture (2:1) containing an internal standard (LPA C14:0). After back extraction with chloroform and water, the centrifuged lower phase was evaporated, redissolved in MeOH and analysed using ESI-MS-MS (directly injected into the ion source) [122].

Im et al. used a modified Matyash method [123] to isolate sphingomyelins (SM) from the plasma of men with abdominal obesity. Plasma was mixed with of 75% MeOH containing BHT and internal lipid standards. After MTBE (1 mL) was added to the mixture, it was shaken, and water was added to separate the phases. The mixture was then centrifuged, and the upper phase was dried and redissolved in of chloroform/MeOH (1:9; *v*/*v*) for liquid chromatography-tandem mass spectrometry (LC–MS/MS) analysis [50].

Wang et al. used a simple method of extraction in the study of the plasma lipidome in adults with obesity. Plasma was mixed, sonicated and incubated with a solution of chloroform/MeOH (2:1) containing internal standards after centrifugated and dried. The extracted lipids were resuspended in butanol and 10 mM NH_4_CHOO in MeOH. After liquid chromatography–electrospay ionization-tandem mass spectrometry (LC–ESI-MS/MS) analysis, they quantified 328 lipid species from 24 lipid classes and subclasses [124].

Misra et al. used untargeted metabolomics analysis of serum in high-fat and high-cholesterol (HFHC) diet-fed baboons. Serum sample extraction was performed by sequential solvent extraction of serum samples initially with an ACN/IPA/water (3:3:2) mixture (1 mL) and later with ACN/water (1:1). The obtained extracts were mixed, dried and subjected to further chemical derivatization (N-methyl-trimethylsilyl-trifluoroacetamide (MSTFA) and N-(t-butyldimethylsilyl)-N-methyltrifluoro-acetamide (MTBSTFA) strategies), which allowed us to quantify 515 metabolites, many of which are involved in lipid metabolism [125].

Wang et al. developed a method to perform plasma lipidomics in spontaneously obese rhesus monkeys, based on cooling a plasma mixture with MeoH/ *n*-hexane with liquid nitrogen, followed by incubation with acetyl chloride for 24 h, adding a K_2_CO_3_ solution, and extracting the obtained methylated FAs with hexane for further analysis using GC-MS. This method allowed the identification, quantification and classification of 143 types of lipids [51].

#### 3.1.1. Serum/Plasma Cholesterol

Dyslipidaemia that develops during obesity is reflected in the levels of both free cholesterol and its individual fractions [42]. To measure indicators such as TC, cholesterol, HDL and TAG in the blood, automated clinical systems based on enzymatic-calorimetric analysis are widely used, while the determination of LDL was not possible until recently. Its value was calculated according to the Friedewald formula [126]. At present, there are many methods for the direct measurement of LDL levels with more accurate results than those obtained by calculation [127].

In the study of cholesterol metabolism in obese people, isotope methods are often used that directly measure the fluxes of [^2^H_2_]- and [U-^13^C]-labelled metabolites [128], and isotope enrichment can be estimated in lipid fractions extracted with organic solvents using the ^13^C to ^12^C ratio, which is measured by gas chromatography continuous-flow isotope ratio-mass spectrometry (which is also used for determining enrichment of [^2^H_2_]) (Finnigan Incos-XL GC-MS) [129]. Cho et al. decided that isotope-kinetic and sterile balance methods are not suitable for large-scale studies and developed their own simple and less-expensive approach for measuring GC-MS serum sterol signatures [82]. For this, serum samples mixed with MeOH and spiked with internal standards were used for hybrid solid-phase extraction-precipitation (H-PPT) and then eluted with MeOH three times. After drying, the pool of eluates was derivatized, and 12 sterol signatures were measured using GC-MS [82].

Recently, studies of fractions and subfractions of cholesterol have been carried out on the basis of size using specialized systems based on gel chromatography. In the work of Lindqvist et al. analysis of lipoprotein fractions during animal bariatric surgery modelling was performed using the Quantimetrix Lipoprint^®^ system (Quantimetrix Corporation, Redondo Beach, CA, USA) [130]. Additionally, Kwon et al. in studies of low-density lipoprotein subfractions in overweight and obese women, also used this method to scan LDL subfractions. This system allowed them to divide LDL into seven subfractions [131]. Doğan et al. also used this system in LDL and HDL subfraction analysis in patients after laparoscopic sleeve gastrectomy [132].

#### 3.1.2. Triglyceride Profiling

The study of blood TG levels is of great interest in the study of obesity because their level quickly responds to dietary changes [133,134] and is associated with insulin resistance [135,136], lipotoxicity [137] and dyslipidaemia [138]. Despite this, studies of the profile of individual FAs in plasma TG are quite rare; this parameter is usually tested in adipose tissue, where TG accumulates [71,139], or in breast milk [140]. Usually, the isolated plasma TAG fraction is examined as one of the components in the study of the whole lipid profile [74,128,141].

Perreault et al. in a study of the inflammatory state of metabolically healthy obese individuals, determined profiles of FAs from serum samples. They measured total FAs and fractionated FAs in PL and TAG fractions. In both cases, isolated lipid fractions were obtained by incubation of samples (45 min) on Silica-G TLC plates (Analtech, Newark, NJ, USA) with petroleum ether, ethyl ether and acetic acid (80:20:1; *v*/*v*/*v*). After that, both the collected PL and TAG lipid bands and the samples for the analysis of total FA were methylated for analysis on a GC with a flame ionization detector (FID) [102].

Much work has been done by Klawitter et al. who compared 4 different approaches for determining the plasma fatty acid desaturation index in response to a carbohydrate-rich diet. Using ultracentrifugation, TLC, SPE, and saponification followed by ultra-high-performance liquid chromatography (UHPLC)-MS for specific fatty acid analysis, they concluded that analysis of specific FAs in the VLDL fraction best reflects the activity of the stearoyl-CoA desaturase 1 (SCD1) metabolic pathway [59]. One of the approaches of UHPLC neutral loss MS was focused on analysing the fatty acid composition of TAG directly from plasma extracts or from plasma VLDL extracts without prior fractionation [59].

#### 3.1.3. Fatty Acid Profiling

The FA profile is a rich source of information on dietary lipid intake and changes associated with obesity. Changes in the FA blood profile affect the production and secretion of cytokines, chemokines and eicosanoids, which can initiate inflammation of the whole body [102]. FFAs released into the blood from adipose tissue during TAG lipolysis may act as a marker of FA adipose tissue composition [142,143].

In our earlier study, we used high-performance liquid chromatography with a laser light scattering detector (HPLC-LLSD) to separate lipid extracts obtained by the Folch method during the identification of cyclopropane FA in the serum of obese people. The resulting FFA, TAG and PL were hydrolysed with 0.5 M KOH in MeOH. After neutralization with 6 M HCl and addition of water (1 mL), triple extraction of FA with *n*-hexane was performed, and the samples were dried again. Thereafter, FAMEs and picolinyl esters of FA were obtained from FFA and FA from the hydrolysis of complex lipids [144]. This method of extraction and hydrolysis followed by FAME synthesis can also be used to determine the levels of odd-chain fatty acids (OCFAs) and branched-chain fatty acids (BCFAs). The study of OCFAs and BCFAs is of interest since changes in these FAs in serum may be associated with insulin resistance in obese patients [145,146].

Kang et al. profiled FAs in the plasma of overweight subjects using a modified version of the Bondia-Pons method [147] to prepare samples for GC analysis. For this, *n*-hexane containing internal standards was added to plasma samples and then mixed with MeOH, and acetyl chloride was slowly added. After incubation 6% potassium carbonate was added to each tube, and after centrifugation, a transparent top layer of *n*-hexane with FAMEs was taken for GC-MS analysis [148]. Later, Lee et al. used this method to determine circulating fatty acid profiles in overweight individuals [149].

Wijayatunga et al. used improved direct synthesis of fatty acid methyl ester to measure serum FAs in patients after bariatric surgery. For this, serum was mixed with MeOH containing internal standard, water solution of 10 N KOH and MeOH. After incubation and shaking, samples were cooled, and 24 N H_2_SO_4_ in water was added and shaken. After that, the obtained FAMEs were extracted with *n*-hexane for subsequent analysis by GC-MS [7].

Aslan et al. studied changes in plasma polyunsaturated FAs (PUFAs) after bariatric surgery. For this, plasma was mixed with an internal standard solution (arachidonic acid (AA)-d8). After a mixture of ACN/37% hydrochloric acid (4:1; *v*/*v*) was added, the samples were hydrolysed, after which extraction was carried out with *n*-hexane. The upper phase, containing FFA, was dried, dissolved in MeOH-water (180:20; *v*/*v*) and filtered for LC-MS/MS analysis [150]. Later, Badoud et al. used this method to determine the profile of FAs in obese individuals. In both works, the authors were able to isolate, extract and quantify 28 FAs [151].

Ma et al. analysed serum FFA profiles to examine the relationship between FFA and the metabolic phenotype of obesity of two ethic groups in China. For this, a sample was mixed with ACN and incubated. After centrifugation, derivatives and 1-ethyl-3-[3-dimethylaminopropyl] carbodiimide were added to improve detection sensitivity, degree of separation, and binding efficiency, which allowed them to quantify 34 types of FFAs in serum [152].

Itariu et al. determined the profiles of FA in plasma phospholipids in severely obese nondiabetic patients. The main lipids were separated by TLC on silica gel plates (Merck) using *n*-hexane/diethyl ether/acetic acid (80:30:1; *v*/*v*) and BHT as the mobile phase and 11,2-dioleoyl-*sn*-glycero-3-phosphocholine (as the standard). After a solution (100 mg of berberine chloride in 100 mL of ethanol) was sprayed, lipid spots were visualized in ultraviolet light. Phospholipids were scraped into glass tubes, followed by methanolysis with a solution of MeOH/toluene (4:1). After 6% K_2_CO_3_ was added, the organic phase was collected and analysed by GC-MS [86].

Given the effect of high plasma NEFA levels on insulin resistance and insulin secretion, Nemati et al. studied changes in NEFA in patients with type 2 diabetes after bariatric surgery [153]. Dole extraction was performed to separate NEFAs. For this, serum was mixed with an internal standard (heptadecanoic acid in IPA and a modified Dole’s mixture containing IPA/n-heptane/phosphoric acid (2 mol/L; 40/10/1; *v*/*v*/*v*). After incubation, n-heptane and water were added, and the samples were mixed and centrifuged. Then, the upper organic layer was dried and dissolved in IPA for analysis by LC-MS, which quantified 5 NEFAs (palmitic, stearic, oleic, palmitoleic and linoleic acid) [153].

Lin et al. described a procedure for FFA analysis in serum samples of patients after bariatric surgery in which a blood serum sample was evaporated in a nitrogen atmosphere and then addedd triheptadecanoin as an internal standard. After drying, the samples were subjected to direct esterification by dry 2.5 M HCl in methanol. The obtained FAMEs were extracted twice with isooctane, and a total of 16 FAs were determined using a GC instrument equipped with a FID [154].

Ramos-Molina et al. used two specific lipid extraction protocols to obtain the serum lipidome in obese subjects after bariatric surgery. Two separate ultrahigh-performance liquid chromatography (UPLC)-MS-based platforms analysing MeOH and chloroform/MeOH serum extracts were combined. In the first protocol, PPT was carried out by adding MeOH to serum. After vortexing and incubation, the supernatants of the samples were centrifuged, dried and restored in MeOH to determine FAs, bile acids (BA), steroids and LPL. A second protocol was used to determine glycerolipids, cholesterol esters, sphingolipids and phospholipids. For this, serum extracts were mixed with sodium chloride (50 mM) and chloroform/MeOH (2:1). After stirring and incubation, the samples were centrifuged, and the organic phase was collected, dried and restored in ACN/IPA (1: 1). Both extraction protocols used internal standards for each class of lipids. After that, two separate UPLC-MS-based platforms were used for analysis, allowing the identification of nearly 300 lipids present in human plasma [155].

Recently, new studies on FAHFAs that are related to diabetes and obesity have appeared [73,79,87,156]. Based on the fact that low concentrations of FAHFA are present in blood serum, López-Bascón et al. developed an automated online SPE LC-MS/MS method for sensitive and selective analysis of FAHFA [87]. Optimization of the SPE protocol included tests with four commercial sorbents (C8, C18, C18HD and Resin SH) packed with the same technology and based on nonpolar interactions. After testing the retention/elution ability of these sorbents using the generic reversed-phase protocol, the greatest retention/elution of FAHFAs was exhibited by C8. This sorbent was used in further optimization of the protocol, which involved varying the composition, volume and flow rate of the tested solvents; the gradient and the elution time [87].

Short-chain fatty acids (SCFAs) are saturated aliphatic FAs with fewer than six carbon atoms that can be produced by the anaerobic intestinal microbiota or by catabolism of branched-chain amino acids. The important role that SCFAs play in homeostasis and data indicating the association of SCFAs with multiple metabolic diseases make them an important object of research [58,157,158]. Other research methods targeting SCFAs are commonly based on extraction with a complex derivatization procedure for subsequent GC–MS analysis [158,159,160]. Zeng and Cao developed a novel LC-MS/MS method using fast derivatization with O-benzylhydroxylamine (O-BHA) and *N*-(3-dimethylaminopropyl)-*N*′-ethylcarbodiimide hydrochloride (EDC) in combination with LLE with dichloromethane for detecting SCFAs in the serum of obese and lean mice [58].

When describing FA profiling, it is important to mention such important metabolites of PUFAs such as oxylipins and endocannabinoids. It has long been known that endocannabinoids are key components of systems that regulate both nutrition and body mass, and oxylipins are also closely associated with obesity due to the wide range of their biological effects [53,161]. In our previous works, the features of sample preparation and methods for the analysis of oxylipins and endocannabinoids in biological samples are described in sufficient detail [60,162]. Compared to other classes of lipids, the analysis of oxylipins can be complicated by their low content in samples, susceptibility to oxidation, and ability to be synthesized de novo during sample preparation and extraction, all of which should be taken into account in the analysis of oxylipins.

Astarita et al. proposed an SPE method to fractionate lipid classes in which in one part of the organic phase after solvent extraction, high-abundance positively charged lipids were analysed by LC/MS. In the second part, the lipids were fractionated according to their relative polarities by serial elution with chloroform/MeOH (9:1 and 1:1; *v*/*v*) mixtures on open-bed silica gel columns (silica gel 60 230–400 mesh). Moreover, for further chromatographic separation, two different octadecyl (C18) columns were used [99]. Later, Argueta et al. used this protocol for the extraction of endocannabinoids from the plasma and jejunum of mice with western diet-induced obesity and their analysis by UPLC-MS/MS [100]. Additionally, Perez et al. used the method of Astarita et al. when studying the endocannabinoid system in plasma, pancreas and jejunum by UPLC-MS/MS in offspring obtained from obese mice fed a Western diet during pregnancy [101].

Ferreiro-Vera et al. developed a fast automatic method for quantitative analysis of oxylipins in serum samples from obese individuals. The approach is based on online SPE–LC–MS/MS method in which a sequence of automatic operations was performed on injected human serum. Further steps included rinsing the cartridge with MeOH, conditioning with water, loading sample into the cartridge with water and, after washing with 20% MeOH in water, switching a valve to elute through the cartridge into the chromatographic column using a mobile phase containing MeOH/water/ACN/acetic acid (76:22:2:0.02; *v*/*v*) [57].

Pickens et al. studied plasma lipid FA profiles in subjects with various BMIs and performed nonesterified plasma PUFA and oxylipins extraction and isolation. Oxilipids were isolated on SPE Phenomenex Strata-X columns (60 mg/3 mL, Phenomenex, Torrance, CA, USA) [163]. Later, Pickens et al. used this method for the extraction and isolation of nonesterified PUFAs and oxylipins [53].

Hernandez-Carretero et al. investigated changes in oxylipins, endocannabinoids, and Cer in mouse plasma after weight loss using a 96-well Ostro™ Pass Through Sample Preparation Plate (Waters Corp, Milford, MA, USA) to remove proteins and phospholipids. [52].

Azar et al. performed LC-MS/MS analysis of endocannabinoids in the serum of patients undergoing bariatric surgery. For this, after PPT with a mixture of acetones and Tris buffer, serum samples were homogenized in a mixture of MeOH and Tris buffer with the addition of an internal standard. The resulting homogenates were extracted with a mixture of chloroform/MeOH (2:1; *v*/*v*), washed three times with chloroform, dried and redissolved with MeOH [164].

Fan et al. investigated the production of unesterified oxylipins and endocannabinoids in mice fed a high-fat diet. To extract oxygenated PUFA metabolites, plasma samples were mixed with antioxidants (BHT/EDTA in MeOH:H_2_O; 1:1) and deuterated surrogates in MeOH. Then, the proteins were precipitated by adding a mixture of MeOH/ACN (1:1). After centrifugation, the supernatants were filtered and quantified using UPLC-MS/MS] [54].

#### 3.1.4. Ceramides and Sphingolipids

Sphingolipids are one of the most complex and structurally diverse families of compounds. Sphingolipids are not only components of biological structures such as membranes and lipoproteins but also highly biologically active compounds that affect dozens of biological processes [165]. Ceramides are sphingolipids that promote insulin resistance and are associated with the distribution of body fat, obesity, and type 2 diabetes [74]. Brozinick et al. used the method of single-phase extraction with MeOH-dichloromethane for the quantitative determination of seven types of Cer in rhesus macaque plasma on a Western-style diet [166].

León-Aguilar et al. used the method described by Croyal et al. [167] to quantify plasma Cer in offspring born to obese women. The method consisted of preparing seven standard dilutions (1, 5, 10, 50, 100, 250, and 500 nM) of 10 types of Cer in MeOH. Then, standard solutions and plasma samples (25 μL) were extracted using the Bligh and Dyer method with the addition of Cer (d18:1/17:0) as an internal standard for further UPLC-MS/MS analysis [76]. Neeland et al. when studying the role of Cer in the development of insulin resistance, used a method based on comparing total acyl carbon content and degrees of saturation between samples and a deuterated internal standard Cer (d18: 1) to identify 13 different types of Cer in plasma by UPLC-MS [168].

Özer et al. were able to quantify five Cer and three SM using successive chloroform/MeOH and chloroform/water extractions before ultrafast liquid chromatography (UFLC)-MS/MS analysis when studying changes in serum SM and Cer after bariatric surgery [169].

### 3.2. Adipose Tissue

In the study of obesity, adipose tissue is more useful than just its basic physiological functions (storage and release of fat); adipose tissue plays an important role in insulin sensitivity and is the site of the synthesis of many hormones and other signaling molecules associated with obesity [71,170,171]. Moreover, adipose tissue is relatively easily available as a research material and can be obtained during bariatric surgery. Most lipid extractions are carried out at RT, which is associated with poor lipid solubility in cold conditions; however, overheating can occur (during homogenization or sonication) and lead to the release of acyl FAs and the formation of lysolipid species [55]. For these reasons (in addition to reducing oxidation), antioxidants (BHT) and metal chelators (EDTA) are added to samples [52,83,103].

Roberts et al. described several approaches based on Folch extraction from white adipose tissue. The described methods, including GC–MS analysis of total fatty acid composition and intact lipid and acylcarnitine profiling by LC–MS, make it possible to obtain wide lipid profiles. At the same time, the authors suggest using SPE with LC–MS/MS analysis for eicosanoids from adipose tissue [90].

Kunešová et al. studied the fatty acid composition of TG in adipose tissue after weight loss. For this, the lipid fraction was extracted from a fat cake obtained during the extraction of RNA and then transmethylated to FAMEs (1 M sodium methoxide; RT; 60 min). After neutralization with 1 M acetic acid, the FAMEs were extracted twice with *n*-hexane, passed through a column (5 × 20 mm) of anhydrous sodium sulfate, mixed together and dried for further analysis on a GC instrument equipped with an FID [172]. Later, Montastier et al. also used this method (lipid fraction extraction from fat cakes produced during RNA extraction) for the analysis of FAs in the adipose tissue of obese women [173].

Hu et al. used a three-phase MTBE/MeOH/water extraction procedure for quantification of FAHFAs in a WAT sample of hamsters fed an HFD. In addition, the samples were subjected to additional removal neutral lipids by subsequent SPE, which allowed the identification of 64 FAHFAs [79].

Okada et al. measured the concentrations of lipid mediators in adipose tissue of obese mice. For this, the samples were extracted using an automated system (RapidTrace Biotage). Samples were pumped onto C18 cartridges, washed with water, *n*-hexane was added, and oxylipins were eluted with methyl formate [85].

Similar to plasma, when the levels of oxylipins are being examined in adipose tissue, additional purification with SPE is usually required. For example, Itariu et al. when studying chronic inflammation of adipose tissue in patients with severe obesity, used SPE (Oasis HLB Extraction Cartridge; Waters) to extract lipid mediators, which was followed by LC-MS [86].

Simple LLE extraction without SPE can also be quite effective in lipidomics. Therefore, Al-Sulaiti et al. successfully used the Bligh and Dyer method to extract lipids for further nontarget LC-MS determination of 76 TAG species from subcutaneous adipose tissue and omental depots from obese individuals [71]. Additionally, using a two-step chloroform/MeOH extraction, Grzybek et al. identified more than 300 lipids in different AT types of mice fed a high-fat diet (a rodent model of obesity) [174]. It is interesting that although all stages of fluid processing were performed using the Hamilton Robotics STARlet robotic platform with the Anti Droplet Control function, the authors encountered difficulties pipetting reproducible amounts of homogenized tissues due to the presence of large amounts of fat droplets when using an aqueous buffer. The problem was solved by homogenization of AT in 50 vol.% ethanol, followed by dilution in pure ethanol [174].

Tomášová et al. described their modification of the Folch lipid extraction method, which solved the problem of the high content of TAG in adipose tissue. They used TLC for separating lipid classes before LC-MS analysis, which allowed them to detect 37 lipids that were below the detection limit without TLC [103]. Similarly, Choromańska et al. applied the previously described method (Bligh and Dyer LLE with TLC on silica gel plates) for TAG, CER and DAG separation in adipose tissue of women with morbid obesity [74].

Depending on the studied lipid class and the selected extraction method, an appropriate organic solvent/mixture of solvents is added during or after homogenization. Mutemberezi et al. used dichloromethane/MeOH/water (8:4:2) extraction with subsequent SPE in LC-MS analysis of oxysterols, Cer, and endocannabinoids involved in obesity and metabolic syndrome [83]. The authors paid special attention to the elimination of the cholesterol oxidation during extraction. To minimize this process, they chose solvents with low prooxidant properties, and to remove cholesterol from the sample, they created an SPE procedure where three different solvent mixtures, *n*-hexane/IPA (99:1; *v*/*v*), *n*-hexane-diethylether (90:10; *v*/*v*) and *n*-hexane-dichloromethane (80:20; *v*/*v*), were tested. Based on their ability to selectively elute cholesterol and their tendency to oxidize cholesterol (5α,6α-epoxycholesterol,27-oxysterol), they chose the *n*-hexane-IPA mixture as the best for this purpose [83].

Serbulea et al. developed a targeted LC-MS approach to analyse oxidized phospholipids (OxPL) in lean and obese mice. OxPL was extracted by a modified Bligh and Dyer method. The method consisted in the fact that the aqueous phase remaining after the first extraction with a chloroform/MeOH mixture was extracted with chloroform, and the organic layer of the second extraction was combined with the first, dried, and resuspended in 300 μL of the mobile phase for further LC-MS analysis. In addition, phospholipids were separated on an EVO C18 column (Kinetex 5 μm; 100 × 4.6 mm; Phenomenex) using a binary gradient [75].

Hanzu et al. when studied visceral (VIS) and subcutaneous (SC) adipose tissue from obese subjects, examined not the tissue itself but the adipose tissue-conditioned medium samples, which were obtained by 24-h incubation of intact fat pads in serum-free medium, and further annotation and identification of metabolites was performed using GC-MS [175].

### 3.3. Liver

Although in the study of obesity, the liver is a pivotal tissue associated with lipid metabolism, similar to adipose tissue, there are few studies describing the extraction and identification of lipids in the liver. Undoubtedly, it is associated with difficulties in obtaining samples from this organ from humans. Most often, liver samples from rodent models of obesity are used. In various studies based on animal model liver tissue, lipids such as oxysterols, endocannabinoids, Cer [83], FAHFAs [79], SM, Cer [176], and glucosylceramides [177] have been studied. As mentioned above, Fan et al. used simple MeOH extraction for the analysis of oxylipins and endocannabinoids from the livers of mice fed a HFD [54].

Pakiet et al. studied the effect of a western diet on mouse brain lipid composition, but they also examined the liver; after a Folch extraction, they used two SPE procedures to separate lipid species [80]. In the first procedure, various eluents were used during SPE with chloroform/IPA (2:1; *v*/*v*) that allowed neutral lipids (NL) to be obtained; diethyl ether with 2% acetic acid (*v*/*v*) was used to elute FFAs, and MeOH was used to extract PLs. After that, neutral lipids were dissolved in *n*-hexane, fractionated on a new SPE cartridge and eluted, and cholesterol esters (with 6 mL *n*-hexane) were discarded. TAG (elution with methylene chloride/diethyl ether/*n*-hexane; 10:1:89; *v*/*v*/*v*), cholesterol (elution with 5% ethyl acetate in *n*-hexane (*v*/*v*), diacylglycerols (DAG) (elution with 15% ethyl acetate in *n*-hexane (*v*/*v*) and monoacylglycerols (MAG) (elution with chloroform-MeOH (2:1; *v*/*v*)) were obtained. After that, the MAG, DAG and TAG fractions were mixed together into a mixture of acylglycerols [80]. Using the second method, from the lipid extracts recovered in chloroform, the following were obtained by extraction: NL (15% ethyl acetate in *n*-hexane (*v*/*v*) was eluted), Cer (chloroform/MeOH; 23:1, *v*/*v*), FFA and α-hydroxy-FFA (5% acetic acid in diisopropyl ether (*v*/*v*)), glycosphingolipids (GSPL) (acetone-MeOH (9:1.35; *v*/*v*), and SM (chloroform/MeOH (2:1; *v*/*v*)). After hydrolysis of all collected lipid fractions and derivatization, the obtained FAMEs were determined using GC-MS [80].

Lytle et al. used chloroform/MeOH (2:1) extraction followed by TLC to fractionate total lipids in a study of steatohepatitis in obese mice; after that, the lipids were saponified, and the FAs were extracted with *n*-hexane and identified using RP-HPLC with a UV detector. Additionally, for some other types of lipids from saponified FAs, FAMEs were prepared, which were extracted in *n*-hexane for subsequent quantification by GC with an FID [104].

Du et al. studied the hepatic expression of sirtuin 5 (SIRT5) in obese mice and determined the liver TAG content. For this, Soxhlet extraction of liver lipids using diethyl ether as a solvent was performed; in addition, to study changes in the size and number of lipid droplets (LD) in hepatocytes, neutral lipids were stained with probe-lipid TOX Green, and haematoxylin and eosin staining was performed [178].

Yetukuri et al. performed lipid profiling in the liver in obese mice with hepatic steatosis. For this, 20–30 mg tissue sample was subjected to chloroform/MeOH extraction with an internal standards mixture (diacylglycerophosphocholine (GPCho) (17:0/17:0), diacylglyceroethanolamine (17:0/17:0), GPCho (17:0/0:0), Cer (d18:1/17:0), and TG (17:0/17:0/17:0)) after which to the separated lower phase, 10 μL of a labelled standard mixture was added, and the sample was analysed by UPLC-MS [179].

Wang et al. when analysing obese mouse liver by shotgun lipidomics, developed an approach that solves the problem of analysing lipids such as LPL that remain in the aqueous phase and are usually discarded after the classical extraction procedure of Bligh and Dyer (because they cannot be directly used for MS analysis due to a high salt content). The method is based on the purification of the aqueous phase solution remaining after LLE extraction. For this purpose, the aqueous phase was loaded onto a Hybrid SPE cartridge that had been twice washed with MeOH; next, lysophosphatidylinositol (LPI), lysophosphatidylserine (LPS) and lysophosphatidylglycerol (LPG) species were eluted with a 10% solution of MeOH in ammonia, and the LPAs were eluted with a solution of 20% ammonia in MeOH. Furthermore, SPE eluents were dried and reduced in MeOH prior to analysis by MS [70].

One of the most comprehensive approaches to liver lipidomics was shown by Garcia-Yaramillo et al. when studying Western diet-induced nonalcoholic steatohepatitis in mice. They used GC analysis of free and saponified FAs converted to FAMES to quantify SFA, MUFA, and ω3, and ω6 PUFA. For quantification of DAG, TAG, PC, phosphatidylserines (PS), phosphatidylinositols (PI), phosphatidylglycerols, PE, LPL and SM, they used an untargeted LC/MS approach. For this, extraction was performed with methylene chloride/IPA/MeOH (25:10:65; *v*/*v*/*v*; -20 °C). In addition, they quantified ω3 and ω6 PUFA and PUFA-derived oxylipins using targeted LC/MS. For this, MeOH extraction with additional SPE on Strata-X columns was used. Both targeted and nontargeted analyses were performed on the same UHPLC system (a Shimadzu Nexera system coupled to a triple time-of-flight (TOF) 5600 mass spectrometer) and the same column (Waters Acquity (UPLC); CSH C18) [98].

In their studies of obesity, Preuss et al. drew attention to the composition of LDs in various tissues, including the liver. To separate LDs from the cytosolic and membrane fractions in homogenates, the authors used 1 h of centrifugation (100,000× *g*; 4 °C), after which LDs formed a clear top layer. Then, lipids from LDs were extracted according to the Folch method. The authors focused on the use of a Centri-Tube slicer (Beckman Coulter, Brea, CA, USA) that increases the purity of the collected LD fraction. Subsequent SPE (Sep Pak Diol Cartridges; Waters, MA, USA) followed by LC-MS/MS analysis allows the determination of diacylglycerols and Cer in all cell fractions [180]. The authors noted that compared with other tissues, the liver had a high level of lipids; therefore, 10 mL of buffer was used to homogenize 20 mg of liver tissue, whereas in the case of muscle and cardiac tissue, 50 mg was homogenized in 500 μL of buffer [180].

### 3.4. Brain

Due to the difficulties of collecting material for research in humans, the study of lipid metabolism in the brain tissue most often occurs with material obtained from animals with obesity induced by a high-energy diet. In addition to difficulties with biopsy, in cases of animals with large brains as in humans, additional difficulties arise due to the heterogeneity of cell populations in grey and white matter, which can lead to different ratios of cell populations in samples [181].

Yang et al. in a study of circulating Cer in HFD-fed mice, used the methodology of Bielawski et al. [182]. After homogenization of frozen tissues with a buffer (0.25 M sucrose, 25 mM KCl, 50 mM Tris, and 0.5 mM EDTA, pH 7.4) in a 1:10 (*w/v*) ratio, homogenate was filtered through layers of gauze. After that, tissue homogenates were spiked with IS solution and extracted twice with IPA/water/ethyl acetate (30:10:60; *v*/*v*/*v*) mixture with subsequent vortexing, sonication and centrifugation. The combined upper layers were dried and reconstituted in the mobile phase for LC-MS analysis [183]. Later, Gao et al. used this approach when exploring ceramide metabolism in the hypothalamus of mice in a model of leptin hypothalamic control of feeding [184].

Rutkowsky et al. performed metabolic analysis of complex lipids in the brains of mice fed a western diet. For this, they used the Matyash protocol [123] based on methyl tert-butyl ether (MTBE) extraction. [185]. Additionally, LLE protocol with MeOH/ethyl acetate in this work was used, for analysis of non-esterified oxylipins and endocannabinoids in the brain was carried out [185].

Rawish et al. performed hypothalamic lipid analyses in mice fed a high-fat diet. For this, they combined several approaches, including using fluorescence microscopy to quantify neutral lipids in the LD hypothalamus (staining LD with lipophilic dye (LD540)) and studying the metabolism of FAs in slices of the hypothalamus using the tracer alkyne oleate; lipidomics analysis of the hypothalamus (analysis of neutral glycerolipids, phosphoglycerol, sphingolipids and acyl-carnitine lipids) was also performed by LC-MS [186].

Kirkham et al. studied the effects of diet on endocannabinoid levels in the rat forebrain and hypothalamus using 5 v of chloroform/MeOH/50 mM Tris HCl (2:1:1) for endocannabinoid extraction from tissue homogenates. After consequent double extraction with 1 v of chloroform, organic phases were pooled, dried and resuspended in chloroform/MeOH (99:1; *v*/*v*) for GC-MS analysis, but before this, the obtained solutions were purified by open bed chromatography on silica and further fractionated by normal-phase high-pressure liquid chromatography (NP-HPLC) on a silica column (Spherisorb S5W; Phase Sep, Queensferry, Clwyd, UK) using a 40 min linear gradient [161].

### 3.5. Skeletal Muscle

Gudbrandsen et al. analysed lipid metabolism in rats after bariatric surgery and extracted FAs from skeletal muscle. They used the Bligh and Dyer method with the addition of heneicosanoic acid as an internal standard. Then, the extracts were methylated in anhydrous MeOH containing 2.5 M HCl (100 °С; 2 h) and extracted twice with isooctane, and methyl esters were quantified using GC with an FID [187].

The most commonly studied classes of muscle lipids in obesity are TAG and CER since the accumulation of these lipids in muscle tissue is associated with the development of IR [188]. The aforementioned Preuss et al. also examined muscles when studying the composition of LDs and determined DAG and Cer in cell fractions [180].

Van Hees et al. when checking the “lipid overflow” hypothesis, did not limit their analysis to TAG content in muscles during the study of IR because they also divided the total amount of lipids obtained after extraction by chloroform-MeOH into FFA, DAG, TAG and PL by TLC before further GC-MS analysis [129].

Laurentius et al. developed a new method to identify and classify FAMEs by GC-MS in HFD-fed rats. At the initial stage, CE, TAG and GPL fractions were extracted from muscle homogenates using tert-butyl methyl ether (90%, tert-BME) and MeOH, and the FFA fraction was extracted using a chloroform/MeOH (2:1) mixture. In the further separation of lipid classes, they used SPE during which they sequentially eluted the CE fraction (elution 1% methyl acetate/*n*-hexane; *v*/*v*) and TAG fraction (elution of 2.5% methyl acetate/*n*-hexane; *v*/*v*), and after the column was washed with acetone, the GPL fraction was eluted with MeOH. FFА were extracted with 80% *n*-hexane/diethyl ether (*v*/*v*) on separate SPE columns, followed by incubation with 1% sulfuric acid in MeOH, addition of 5% sodium chloride, and three washes of the *n*-hexane layer with water [97].

One of the latest approaches developed by Eum et al. allows the identification of hundreds of individual lipid species in the muscle tissue of mice with HFD [78]. A two-stage extraction method was performed using MTBE for the first extraction and subsequent secondary extraction of lipids from the lower aqueous layer with MeOH. After mixing, the organic layers were dried and reduced with a mixture of chloroform/MeOH (1:9; *v*/*v*) for subsequent nanoflow ultrahigh-performance liquid chromatography with tandem mass spectrometry (nUHPLC-ESI-MS/MS) analysis [78].

### 3.6. Heart

The study of lipid metabolism in heart tissue is of interest primarily due to the strong influences of obesity and a high-fat diet, particularly the influences on the phospholipid composition of mitochondrial membranes of cardiomyocytes [189]. Due to the unique lipid composition of mitochondrial membranes, they are very sensitive to high levels of FAs and their oxidation products in the blood [189].

Harmancey et al. when studying changes in the composition of cardiac acyl-CoA in obese rats, used the Bligh and Dyer method to extract cardiac lipids from heart tissue; then, extraction was repeated three times to complete lipid recovery for further quantification of Cer and diacylglycerols by HPLC-UV [190]. When the cardiac ceramide content in rats fed a high-fat diet was studied, the Bligh and Dyer method was also used to extract total cardiac lipids [191]. Later, they used the extraction method described by Merrill et al. [165], which is based on extraction with a mixture of MeOH and chloroform, followed by sonication (48 °C; overnight), which is necessary for the extraction of sphingolipids, followed by incubation (37 °C; 1 h) to remove interfering glycerolipids (in particular PC). After another extraction with chloroform, ceramide species were detected by LC-MS [165].

The abovementioned protocol of Pakiet et al. with the use of LLE and two SPE methods for extracting lipids from the brains of mice was also successfully used to extract lipids from the hearts of mice fed a high-fat diet [192].

### 3.7. Other Biological Materials

#### 3.7.1. Urine

Feng et al. used an approach based on UPLC-MS to study the effects of HFD on glycolipid metabolism in young obese men. Metabolomic profiling was performed on urine samples that were centrifuged (14,000× *g*; 4 °C; 10 min) and then analysed using UPLC-QTOF-MS/MS [193]. However, to obtain a more complete urinary lipid profile, extraction with organic solvents was necessary [197].

#### 3.7.2. Saliva

Araujo and Santos used high performance liquid chromatography–diode array detector (HPLC–DAD) Shimadzu analytical system to show the possibility of using saliva as a biomarker. The authors used chloroform/MeOH extraction for measurements of cholesterol and 7-ketocholesterol, and MeOH/IPA extraction was used for measurements of 25-hydroxyvitamins D2 and D3. The authors claim that saliva, with its simplicity and non-invasiveness of collection, can be an important source of markers of nutritional status [194]. This may indeed be true since the study of changes in the metabolism of vitamin D associated with obesity in adipose tissue, in addition to requiring invasive intervention, requires complex MTBE extraction followed by SPE for further liquid-chromatography high-resolution tandem mass spectrometry (LC-HRMS/MS) determination of 25-hydroxyvitamin D and 1,25-dihydroxyvitamin D in adipose tissue [198].

#### 3.7.3. Follicular Fluid

Ruebel et al. used two untargeted metabolomics approaches for primary metabolism analyses and metabolomics assessment of complex lipids in the study of follicular fluid (FF) in obese women. The first approach included GC-MS analysis of FF samples after extraction and derivatization by silylation/methyloximation [195]. During the second approach after MTBE extraction, FF samples were analysed using CSH-ESI QTOF MS/MS; negative ion MS was used for FFA and PI analysis, while PC, LPC, PE and PS were analysed in positive ion mode [195].

#### 3.7.4. Faecal samples

Regarding nonstandard approaches, it is worth mentioning that de la Cuesta-Zuluaga et al. developed a method for determining SCFAs in faecal samples from obese subjects. They determined volatiles using a CTC Combipal 3 autosampler in HS/SPME mode equipped with a grey fibre (Carboxen/DVB/PDMS–ref. SU57329U; Supelco) in water extracts heated to 80 °C, which was followed by desorption of the fibre (at 250 °C) and GC-MS analysis [196].

## 4. Conclusions

Using lipidomics in the study of obesity is very important to understanding the regulatory and diagnostic value of changes in the lipidome associated with obesity. Depending on the studied tissue, the role of changes in specific lipids may have different levels of importance (CER and TAG in muscles, fatty acid composition of TG in adipose tissues, etc.). Despite the fact that new modifications of extraction methods such as those of Bligh and Dyer and new methods (e.g., MTBE) are constantly emerging, none of the extraction methods allows for quantitative extraction of all types of lipids due to the huge differences in their chemical characteristics. This may explain the great popularity of the targeted approach and the SPE method, which can reduce lipid degradation during extraction and can also be automated. At the same time, LLE using phase separation is a more suitable method for nontarget lipidomics, where fractionation is not required. Thus, the study of changes in lipid metabolism in obesity contributed to the development of new methods for sample preparation, extraction and quantification, not only to improve the accuracy and sensitivity of existing methods but also to develop methods for detecting new specific markers among a huge variety of lipid compounds.

## Figures and Tables

**Table 1:** Sample preparation methods for lipidomics approaches used in the studies of obesity.

Lipid Class(es)	Matrix	Sample Preparation Method			Analysis Method	References
		Pre-Preparation	Extraction Method	Derivatization Step		
PL	Plasma	-	modified Folch method	sodium methoxide—FAME	GC	Blewett et al. [121]
FFA, TAG	Plasma	-	Bligh and Dyer’s method	14% BF3 - MeOH - FAME	GC-MS	Choromańska et al. [74]
LPAs	Plasma	-	hydrochloric acid + MeOH/chloroform (2:1)	-	ESI-MS –MS	Yoon et al. [122]
SM	Plasma	MeOH	modified Matyash method	-	LC-MS/MS	Im et al. [50]
lipidomic profile (328 lipid species from 24 lipid classes: dhCer, Cer. MHC, DHC, THC, GM3, SM, PC, PC(0), PC(P), LPC, PE, PE(0), PE(P), LPE, PI, LPI, PS, PG, CE, COH, DG, TG)	Plasma	MeOH	chloroform/MeOH (2:1)	-	LC ESI-MS/MS	Wang et al. [124]
untargeted metabolomics analysis /lipidomic profile? (515 metabolites)	Serum	-	ACN: isopropanol: water (3:3:2)	MSTFA + MTBSTFA	2D GC-ToF-MS	Misra et al. [125]
lipidomic profile (143 lipid species from lipid classes: FA, FFA, PC, PE, PI, PS, PG, LPC, LPA, SM)	Plasma	-	MeOH/n-hexane (4:1)	acetyl chloride + 6% K_2_CO_3_	GC- FID/MS	Wang et al. [51]
sterols	Serum	MeOH	solid-phase extraction (hybrid solid-phase extraction-precipitation (H-PPT) cartridge)	MSTFA/ammonium iodide (NH 4 I)/dithioerythritol (DTE) (500:4:2)	GC-MS	Cho et al. [82]
total FAs + circulating PL, TG	Serum	-	chloroform/MeOH (2:1)	methylation (100 °C; 1.5 h)	GC- FID	Perreault et al. [102]
FFA, TAG, PL	Serum	-	Folch method	10% BF3 - MeOH	GC–MS	Śledziński et al. [144]
SFA, MUFAs, PUFAs	Plasma	-	MeOH	acetyl chloride + 6% K_2_CO_3_	GC-MS	Kang et al. [148]
MCFAs, NEFAs	Serum	MeOH	-	10M KOH in MeOH + 24 N H2SO4	GC-MS	Wijayatunga et al. [7]
PUFAs	Plasma	ACN/37% hydrochloric acid (4:1)	n-hexane	-	LC-MS/MS	Aslan et al. [150]
MUFAs, PUFAs, OCFAs	Serum	ACN	chloroform/MeOH (2:1)	-	UHPLC-MS	Ma et al. [152]
PUFAs	Plasma	MeOH	-	acetyl chloride + 6% K_2_CO_3_	LC-MS	Itariu et al. [86]
NEFAs	Plasma	-	Dole extraction	-	LC-MS	Nemati et al. [153]
MUFAs, PUFAs, SFA	Serum	-	chloroform/MeOH (2:1)	HCl in MeOH	GLC-FID	Lin et al. [154]
FAs, bile acids (BA), steroids, LPL, glycerolipids, cholesterol esters, SPL, PL	Serum	For FAs, bile acids (BA), steroids and LPL - MeOH	For glycerolipids, cholesterol esters, sphingolipids and phospholipids - NaCl + chloroform/MeOH (2:1)	-	UPLC-MS	Ramos-Molina et al. [155]
	Serum	-	chloroform/MeOH (2:1)	-	UHPLC-MS	Ramos-Molina et al. [155]
FAHFAs	Serum	MeOH	solid-phase extraction (hysphere C8 cartridges)	-	LC-MS/MS	López-Bascón et al. [87]
SCFAs	Plasma	MeOH	dichloromethane	0.1 M O-benzylhydroxylamine (O-BHA) in MeOH and 0.25 M N-(3-dimethylaminopropyl)-N′-ethylcarbodiimide hydrochloride (EDC) in MeOH	LC-MS/MS	Zeng and Cao [58]
MAG, FAE, oxFAE, oxMAG, FA, oxFA, DAG, TAG, PE, PI, NAPE, LNAPE, PC	Plasma	-	Chloroform	-	LC/MS	Astarita et al. [99]
endocannabinoids	Plasma	-	Chloroform	-	UPLC-MS/MS	Argueta et al. [100]
endocannabinoids	Plasma	-	Chloroform	-	UPLC-MS/MS	Perez et al. [101]
oxylipins	Serum	-	solid-phase extraction (C18 cartridges)	-	online SPE–LC–MS/MS	Ferreiro-Vera et al. [57]
nonesterified PUFAs and oxylipins	Plasma	MeOH + formic acid	solid-phase extraction (Strata-X)	-	UHPLC-MS/MS	Pickens et al. [163]
oxylipins, endocannabinoid, Cer	Serum	IPA with 10 mM ammonium formate + 1% formic acid	-	-	UPLC-MS/MS	Hernandez-Carretero et al. [52]
endocannabinoids	Serum	acetones + Tris buffer (50 mM, pH 8.0)	chloroform/MeOH (2:1)	-	LC-MS/MS	Azar et al. [164]
unesterified oxylipins, endocannabinoids	Plasma	MeOH/ACN (1:1)	solid-phase extraction (BEH C18 colum)	-	UPLC-MS/MS	Fan et al. [54]
Cer	Plasma	-	Bligh and Dyer method	-	UPLC-MS/MS	León-Aguilar et al. [76]
Cer, SM	Serum	-	chloroform/MeOH (2:1)	-	(UFLC)-MS/MS	Özer et al. were [169]
FA	Adipose tissue	-	MeOH/chloroform (2:1)	10 % BF3 - MeOH	GC–MS	Roberts et al. [90]
acylcarnitines	Adipose tissue	-	MeOH/chloroform (2:1)	-	LC–MS	Roberts et al. [90]
SFA, MUFAs, TFA, PUFAs	Adipose tissue	-	*n*-hexane	sodium methoxide	GC–FID/MS analysis	Kunešová et al. [172]
FAHFAs	Adipose tissue	-	MTBE/MeOH/water	-	UPLC-MS/MS	Hu et al. [79]
oxylipins	Adipose tissue	MeOH	RapidTrace Biotage	-	LC-MS-MS	Okada et al. [85]
TAG	Adipose tissue	-	Bligh and Dyer method	-	LC-MS	Al-Sulaiti et al. [71]
more than 300 lipid species from lipid classes: CL, Cer, ST. HexCer, LPA, LPC, LPE, LPG, LPI, LPS, SM, TAG, CE, DAG, PA, PC, PE, PG, PI, PS	Adipose tissue	-	two-step chloroform/MeOH extraction	-	MS	Grzybek et al. [174]
TAG, MAG, DAG, LysoPC, PC, LysoPE, PE, Cer, SM, PI, PS, FA	Adipose tissue	-	modified Folch method	-	LC-MS	Tomášová et al. [103]
oxysterols, Cer, endocannabinoids	Adipose tissue	-	dichloromethane/MeOH/water (8:4:2) + solid-phase extraction (C18 colum)	-	LC-MS	Mutemberezi et al. [83]
OxPL	Adipose tissue	-	chloroform/MeOH (3:1) + BHT	-	LC-MS	Serbulea et al. [75]
MAG, DAG, TAG, NL, Cer, FFA, GSPL, SM	Liver	-	Folch extraction	10% BF3 - MeOH	GC-MS	Pakiet et al. [80]
STA, MUFA, PUFA	HepG2 cells	-	chloroform/MeOH (2:1) + BHT	hexane + 0.05% BHT	GC- FID	Lytle et al. [104]
Cer, SM, GPCho, GPEtn, GPSer, GPA, GPGro, DG, TG	Liver	-	chloroform/MeOH (2:1)	-	UPLC-MS	Yetukuri et al. [179]
LPL (LPS, LPA, LPI, LPG, LPC, LPE)	Liver	4% formic acids in MeOH	modified method of Bligh and Dyer + solid-phase extraction (HybridSPE cartridge)	-	ESI-MS	Wang et al. [70]
SFA, MUFA, PUFA	Liver	-	chloroform:MeOH (2:1) plus 1 mM BHT	1% H2SO4 in MeOH	GC–FID	Garcia-Yaramillo et al. [98]
PUFA, PUFA-derived oxylipins	Liver	MeOH	solid-phase extraction (Strata-X)	-	targeted UPLC-TOF-MS/MS	Garcia-Yaramillo et al. [98]
DAG, TAG, PC, PS, PI, PG, PE, LPL, SM	Liver	-	methylene chloride/IPA/MeOH (25:10:65)	-	untargeted UPLC-TOF-MS/MS	Garcia-Yaramillo et al. [98]
DAG, Cer	Liver	-	Folch method + solid-phase extraction (Sep Pak Diol Cartridges)	-	LC-MS/MS	Preuss et al. [180]
	Brain	-	IPA/water/ethyl acetate (30:10:60)	-	LC-MS	Yang et al. [184]
non-esterified oxylipins, endocannabinoids, PUFAs	Brain	MeOH	MTBE	-	UHPLC-QTOF-MS	Rutkowsky et al. [185]
Cer, DG, ClcCer, LPC, PC, PE, FA, PI, SM	Brain	-	MeOH/ethyl acetate	-	CSH-ESI QTOF MS/MS	Rutkowsky et al. [185]
endocannabinoid	Brain	-	chloroform/MeOH/50 mM Tris HCl (2:1:1)	MSTFA + 1% trimethylchlorosylane	GC-MS	Kirkham et al. [161]
SFA, MUFA, PUFA	Skeletal muscle	-	Bligh and Dyer method	anhydrous MeOH containing 2.5 M HCl (100 °С; 2 h)	GC-FID	Gudbrandsen et al. [187]
FFA, DAG, TAG, PL	Skeletal muscle	-	chloroform/MeOH (2:1)	14% BF3 - MeOH	GC-MS	Van Hees et al. [129]
CE, TAG, GPL	Skeletal muscle	-	for CE, TAG and GPL fractions - tert-butyl methyl ether (90%, tert-BME) and MeOH; for FFA fraction chloroform/MeOH (2:1) + solid phase extraction	2 M sodium methoxide solution	GC-MS	Laurentius et al. [97]
LPC, LPE, PI, PG, Cer, PC, PE, PS, TG, HexCer, SM	Skeletal muscle	-	two-stage extraction method using MTBE/MeOH	-	nUHPLC-ESI-MS/MS	Eum et al. [78]
DAG, Cer, acyl-CoA	Heart	-	Bligh and Dyer method	-	HPLC-UV	Harmancey et al. [190]
sphingolipids	Heart	-	MeOH/chloroform	-	LC-MS/MS	Merrill et al. [165]
SFA, MUFA, PUFA	Heart	-	chloroform/MeOH (2:1) + solid-phase extraction (Strata)	10% BF3—MeOH	GC–MS	Pakiet et al. [192]
SM, PC, PE, PG, PI, PS	Urine	-	ACQUITY UPLC HSS-T3 C_18_ column	-	UPLC-QTOF-MS/MS	Feng et al. [193]
Cholesterol, 7-ketocholesterol	Saliva	-	for cholesterol and 7-ketocholesterol chloroform/MeOH (2:1); for 25-hydroxyvitamins D2 and D3 MeOH/IPA	-	HPLC-DAD	Araujo and Santos [194]
FFA, PI, PC, LPC, PS, PE, TG	Follicular fluid and serum	-	IPA/acetonitrile/water (3:3:2) or MTBE	MSTFA + 1% TMCS	GC-MS	Ruebel et al. [195]
FFA, PI, PC, LPC, PE, PS	Follicular fluid and serum	-	IPA/acetonitrile/water (3:3:2) or MTBE	-	untargeted CSH-ESI QTOF MS/MS	Ruebel et al. [195]
SCFAs	Faecal samples	-	-	CTC Combipal 3 autosampler in HS/SPME mode equipped with a gray fibe	GC-MS	Cuesta-Zuluaga et al. [196]

2D GC-ToF-MS: two-dimensional gas chromatography time-of-flight mass spectrometer; ESI-MS–MS: turbo electrospray ionization tandem mass spectrometry; GC- FID/MS: gas chromatography-flame ionization detector-mass spectrometer; GC-FID: gas chromatography-flame ionization detection; GLC-FID: gas liquid chromatography-flame ionization detector; HPLC-UV: high-performance liquid chromatography with UV-detection; LC-HRMS/MS: liquid-chromatography high-resolution tandem mass spectrometry; LC-MS-MS liquid chromatography with tandem mass spectrometry; nUHPLC-ESI-MS/MS: nanoflow ultrahigh performance liquid chromatography with tandem mass spectrometry; UHPLC/Q-TOF-MS: ultra-high performance liquid chromatography-quadrupole time-of-flight mass spectrometry; UPLC-QTOF-MS: ultra-performance liquid chromatography quadrupole time-of-flight mass spectrometry; UPLC-TOF-MS/MS: ultra-high performance liquid chromatography coupled to a Triple time-of-flight mass spectrometer; PL: phospholipids; FFA: free fatty acids; TAG: triacylglycerol; LPA: lysophosphatidic acid; SM: sphingomyelin; dhCer: dihydroceramide; Cer: ceramides; MHC: monohexosylceramide; DHC: dihexosylceramide; THC: trihexosylceramide; GM3:ganglioside; PC: phosphatidylcholine; PC(0): alkylphosphatidylcholine; PC(P): phosphatidylcholine plasmalogen; LPC: lysophosphatidylcholine; PE: phosphatidylethanolamine, PE(0): akylphosphatidylethanolamine; PE(P): phosphatidylethanolamine plasmalogen; LPE: lysophosphatidylethanolamine; PI: phosphatidylinositol; LPI: lysophosphatidylinositol, PS: phosphatidylserine; PG: phosphatidylglycerol; CE: chofesterol ester; COH: free cholesterol; DG: diacylglycerol; TG: triacylglycerol; FA: fatty acid; SFA: saturated fatty acid; MUFAs: monounsaturated fatty acids; PUFAs: polyunsaturated fatty acids; NEFAs: non-esterified fatty acids; OCFAs: odd-carbon fatty acids; SPL: sphingolipids; FAHFAs: fatty acid esters of hydroxy fatty acids; SCFAs: short-chain fatty acids; MAG: monoacylglycerol; FAE: fatty-acid ethanolamides; oxFA: oxygenated FAE; oxMAG: oxygenated MAG; oxFA: oxygenated fatty acids; NAPE: *N*-acyl-phosphatidylethanolamine; LNAPE: lyso-NAPE; TFA: trans fatty acids; CL: cardiolipin; ST: cholesterol; HexCer: hexosylceramide; LPG: lysophosphatidylglycerol; LPI: lysophosphatidylinositol; LPS: lysophosphatidylserine; PA: Phosphate; LysoPC: Lysophosphatidylcholines; LysoPE: Lysophosphatidylethanolamines; OxPL: oxidized phospholipids; NL: neutral lipids; GSPL: glycosphingolipids; GPCho: diacylglycerophosphocholine; GPEtn: glycerophosphoethanolamines; GPSer: glycerophosphoserine; GPGro: Glycerophosphoglycerol; GlcCer: Glucosylceramides; Acyl:CoA:dihydroxyacetone phosphate acyltransferase; CSH-ESI QTOF MS/MS: charged-surface hybrid column-electrospray ionization quadrupole time-of-flight tandem mass spectrometry.
